# Validation of the Japanese version of the revised version of the compound psychological capital scale (CPC-12R)

**DOI:** 10.3389/fpsyg.2022.1053601

**Published:** 2023-01-18

**Authors:** Megumi Ikeda, Kai Hatano, Satoshi Tanaka, Jun Nakahara

**Affiliations:** ^1^Institute of Social Science, The University of Tokyo, Tokyo, Japan; ^2^Graduate School of Sustainable System Science, Osaka Metropolitan University, Osaka, Japan; ^3^College of Business, Rikkyo University, Tokyo, Japan

**Keywords:** psychological capital, validation, compound psychological capital scale, measurement invariance, Japanese employees

## Abstract

This study developed a Japanese version of the Revised Version of the Compound Psychological Capital Scale (CPC-12R) and tested its reliability and construct validity. The participants were 1,000 young adults (500 university students and 500 employees) recruited through an internet survey. Confirmatory factor analysis showed that the four first-order factors (hope, optimism, resilience, and self-efficacy) and one second-order factor (PsyCap) model of the previous study is appropriate for the Japanese context. In addition, Cronbach’s *α* and omega-higher-order of CPC-12R were sufficient. The measurement invariance analysis suggested sufficient scalar invariances for the employees and university students and across genders. The Japanese version of the CPC-12R had moderate positive correlations with job satisfaction, work engagement, conscientiousness, and extraversion, as well as a moderate negative correlation with negative emotionality. These findings provide evidence for sufficient reliability and construct validity of the Japanese version of the CPC-12R.

## 1. Introduction

In the present uncertain and rapidly changing business environment (Bennett and Lemoine, 2014), companies seeking to gain a competitive advantage need to enhance employees’ psychological capital (PsyCap; Lorenz et al., 2016; Kim et al., 2017; Vilariño del Castillo and Lopez-Zafra, 2021). PsyCap is an individual’s positive psychological state consisting of hope, optimism, resilience, and self-efficacy (Luthans et al., 2007a); it specifically refers to an individual’s appraisal of the circumstances and potential for success based on motivated effort and perseverance (Luthans et al., 2007b). In times of change, human capital, such as employees’ knowledge, skills, and abilities, can become obsolete (Lorenz et al., 2016; Kim et al., 2017; Vilariño del Castillo and Lopez-Zafra, 2021). PsyCap encourages employees to adapt to such changes and continuously update their necessary competencies, thus giving the company a competitive advantage (Lorenz et al., 2016; Kim et al., 2017; Vilariño del Castillo and Lopez-Zafra, 2021).

PsyCap concept emerged during the incorporation of positive psychology ideas into organizational behavior (Luthans and Avolio, 2009). PsyCap is a higher-order concept consisting of four sub-dimensions: hope, optimism, resilience, and self-efficiency (Luthans and Youssef-Morgan, 2017). Hope means moving toward a goal while changing the goal as needed; optimism means making positive attributions; resilience means overcoming difficult situations; and self-efficacy means confidence in one’s ability in trying to accomplish a challenging task (Luthans et al., 2007b). PsyCap has a stronger relationship with performance and job satisfaction than its four sub-dimensions (Nolzen, 2018). Therefore, prior research focused on PsyCap, which is a higher-level concept, rather than dealing with the sub-dimensions separately.

Empirical studies provide evidence that PsyCap benefits companies. First, it promotes individual performance and desired organizational behavior. For example, Avey et al. (2011) showed a positive relation between PsyCap and employees’ performance, job satisfaction, engagement, and organizational citizenship behavior. Second, it contributes to not only individual attitudes and performance but also group-level performance. Mathe et al. (2017) found that collective PsyCap positively impacts unit revenue, and the effect is fully mediated by service quality and customer satisfaction.

Thus, while the PsyCap concept has been developed in the job domain, its use has recently been extended to other research fields. For example, studies on college students showed that PsyCap is positively associated with college students’ work volition and well-being (Cheung et al., 2020; Poots and Cassidy, 2020). In addition, a study on refugees reported that PsyCap contributes to refugees’ job search self-efficacy *via* career adaptability (Pajic et al., 2018). These studies also showed the effectiveness of PsyCap in improving people’s performance and well-being outside of work (e.g., Lorenz et al., 2016).

The Psychological Capital Questionnaire (PCQ; Luthans et al., 2007a,b) is often used to measure PsyCap. PCQ is acknowledged as the standard measure for PsyCap (e.g., Dawkins et al., 2013; Newman et al., 2014; Nolzen, 2018). This is a 24-item self-report measure, although a short version (PCQ-12) has also been developed and is often used (Luthans and Youssef-Morgan, 2017). The PCQ-24 and PCQ-12 have been translated into French, Portuguese, and Chinese besides English (e.g., Cid et al., 2020; Choisay et al., 2021). The PCQ-12 has been reported to have the same factor structure as the original scale in the samples from Brazil, China, Germany, India, Italy, Mexico, Poland, South Africa, Sweden, Turkey, the UK, and the US (Wernsing, 2013).

Although the PCQ is a widely used measure, it has a disadvantage as it is limited to work situations. Specifically, the questionnaire includes words such as “management” and “company’s strategy,” making it difficult to answer for those not currently working.

In contrast, the Compound Psychological Capital Scale (CPC) and its revised version (CPC-12R) are PsyCap measures that are not limited to workplace situations (Lorenz et al., 2016; Dudasova et al., 2021). Both the CPC and CPC-12R are self-report measures consisting of 12 items. The CPC-12R changes the resilience items of the CPC to match the definition of resilience more closely (Dudasova et al., 2021). This scale allows measurement of PsyCap outside work situations. Specifically, the CPC is used to examine the relationship between PsyCap and work motivation in college students and between job search self-efficacy and PsyCap (Pajic et al., 2018; Cheung et al., 2020).

The CPC and CPC-12R are composed four first-order factors (hope, optimism, resilience, and self-efficacy) and one second-order factor (PsyCap) in terms of factor structure (Lorenz et al., 2016; Dudasova et al., 2021). The CPC has also been validated; it has moderate positive correlation with the following: PCQ, work engagement, job satisfaction, conscientiousness, and extraversion (Lorenz et al., 2016). A moderate negative correlation between CPC and negative emotionality has also been shown (Lorenz et al., 2016).

In Japan, PsyCap research has not progressed. Some researchers have studied PsyCap among employees (e.g., Hattori, 2021; Oto, 2021). For example, Hattori (2021) found that the amount of PsyCap is positively correlated with the likelihood of being certified as a star employee. At the same time, no studies have been conducted on individuals who are currently not working.

One reason for the few PsyCap studies in Japan may be the lack of development of a Japanese version of the PsyCap scale that is not limited to the job domain. Therefore, developing a Japanese version of the CPC-12R could contribute to expanding PsyCap research in Japan beyond the management research area.

The present study, therefore, aimed to verify the reliability and construct validity of the Japanese version of the CPC-12R. We implemented the following three steps of reliability and construct validation. First, we performed confirmatory factor analysis (CFA) to examine whether the Japanese version of the CPC-12R had the same factor structure as that in previous studies (Dudasova et al., 2021). Second, to examine whether the same scale could be used for different people in Japan, we assessed measurement invariance across groups of different genders and occupations (employees and university students). Prior research did not examine whether measurement invariance exists across groups of different occupations. However, given that the PsyCap concept originated in organizational behavior research, measurement invariance between employees and non-employees may not be acceptable. Therefore, we examined measurement invariance by occupation (employees and university students). Third, to examine construct validity, we tested the hypothesis that the Japanese version of the CPC-12R is positively correlated with work engagement, job satisfaction, conscientiousness, and extraversion, as well as negatively correlated with negative emotionality.

## 2. Materials and methods

### 2.1. Procedure

The current study was based on an internet survey conducted by an online survey company, Cross Marketing Inc., in November 2021. The survey included university students and young employees (in their 20s) registered with the above company. Data were collected from 1,000 persons, 500 each for university students and young employees. In both surveys, the allocation was 55% men and 45% women, in line with the Japanese working population. Moreover, regarding employment status in the young employee survey, there were 63% full-time employees and 37% part-timers, also in line with the Japanese workforce.

The Ethics Committee of Osaka Prefecture University approved this study. The participants provided informed consent, and they could stop responding at any time. The survey participants answered the questionnaire anonymously and were paid approximately JPY 50 by the internet monitoring company.

### 2.2. Participants

The mean age of the 1,000 participants was 23.46 years [standard deviation (SD) = 3.37, age range: 18–29 years], and 54.2% were men. In terms of residence, 35.8% lived in the Tokyo metropolitan area, 15.8% in the Kansai area, and 10.5% in the Chukyo area. The remaining 37.9% lived in rural areas.

In the employee survey (500 respondents), 62.8% were full-time employees, and 37.2% were part-time employees. Their average age was 26.12 years (SD = 2.34), and 53.4% of the participants were men. Regarding the length of employment, 19.8% had been with the company for less than 1 year, 29.8% for 1–3 years, 22.2% for 3–5 years, and 28.2% for more than 5 years. In addition, 33.8% lived in the Tokyo metropolitan area, 13.4% in the Kansai area, and 11.8% in the Chukyo area. The remaining 41.0% lived in rural areas.

In the university student survey (500 respondents), 20.6% of the students were first-years, 20.2% were second-years, 22.6% were third-years, 34.6% were fourth-years, and 2% were fifth-years or above. Their average age was 20.79 years (*SD* = 1.74), and 55.0% of the participants were men. In addition, 37.8% lived in the Tokyo metropolitan area, 18.2% in the Kansai area, and 9.2% in the Chukyo area. The remaining 34.8% lived in rural areas.

### 2.3. Measures

#### 2.3.1. CPC-12R

The CPC-12R of Dudasova et al. (2021) was used. It was translated into Japanese by the first author, and then the Japanese version of the CPC-12R was back-translated into English by a bilingual graduate student. The back-translated version was compared with the original scale, and revisions were repeated until the expressions were consistent. An example item is, “If I should find myself in a jam, I could think of many ways to get out of it” (see Appendix). Items were rated on a five-point rating scale ranging from 1 (*completely untrue*) to 6 (*completely true*).

#### 2.3.2. Job satisfaction

We used the four items of Kodama’s (2011) scale. An example item is “I am satisfied with my current job.” Items were rated on a five-point rating scale ranging from 1 (*completely disagree*) to 5 (*completely agree*).

#### 2.3.3. Work engagement

We used nine items from Shimazu et al.’s (Shimazu et al., 2008) Japanese version of the Utrecht Work Engagement Scale (Schaufeli et al., 2006). An example item is, “At my work, I feel that I am bursting with energy.” Items were rated on a seven-point rating scale ranging from 1 (*never*) to 7 (*always*).

#### 2.3.4. Personality

To measure the personality traits of conscientiousness, extraversion, and negative emotionality, we used 18 items from the Big Five Inventory–2 Short Form (Soto and John, 2017). Herein, extraversion consists of six items (e.g., “I am someone who is dominant and acts as a leader”), conscientiousness consists of six items (e.g., “I am someone who is reliable and can always be counted on”), and negative emotionality consists of six items (e.g., “I am someone who worries a lot.”). Items were rated on a five-point rating scale ranging from 1 (*completely disagree*) to 5 (*completely agree*).

### 2.4. Data analysis

First, we conducted confirmatory factor analyses to examine the factor structure. Specifically, we examined the same factor structure as in previous studies (Dudasova et al., 2021): four first-order factors (hope, optimism, resilience, and self-efficacy) and one second-order factor (PsyCap). We also examined other alternative models (four-and one-factor models). We used the following goodness-of-fit indices: root mean square error of approximation (RMSEA), comparative fit index (CFI), and standardized root mean square residual (SRMR). The cutoff values were RMSEA ≤0.08, CFI ≥ 0.95, and SRMR ≤0.08 (Hu and Bentler, 1999; Acock, 2013). To check reliability, in addition to Cronbach’s α, we also checked McDonald’s ω and the omega-higher-order (*ω*_ho_) according to Flora (2020).

Second, we examined measurement invariance across occupations (university students and employees) and genders. Specifically, we examined configural (whether different samples exhibited the same factor structure), metric (whether factor loadings were equivalent across groups), and scalar (whether intercepts were equivalent across groups) invariances. Given that the likelihood-ratio test is affected by sample size, we focused on ΔCFI and ΔRMSEA as goodness-of-fit indices (cutoff values below 0.01 and 0.015; Chen, 2007). The method of examining measurement invariance of the second-order factor model was based on Rudnev et al. (2018). Specifically, the metric invariance of the first-order factors was examined before examining the metric invariance of the second-order factors, and the scalar invariance of first-order factors was examined before examining the scalar invariance of the second-order factors (Rudnev et al., 2018).

Third, to examine construct validity, we calculated correlation coefficients between CPC-12R and job satisfaction and work engagement using data from employees only, as well as between CPC-12R and personality using data from employees and students combined.

CFA and measurement invariance were performed with the R-package lavaan 0.6–12 (Rosseel, 2012). The parameter estimates were identified using maximum likelihood estimation with robust standard errors. Construct validity was examined in StataIC16.

## 3. Results

### 3.1. CFA and reliability

Table 1 presents results of validating the second-order factor model, as in the previous study, and the alternative models (one-and four-factor models). The results showed that goodness of fit of the one-factor model was *χ*^2^ = 487.621, df = 54, *p* < 0.001, RMESEA = 0.121, CFI = 0.896, and SRMR = 0.052; thus, it did not meet the cutoff criteria. In contrast, goodness of fit for the second-order model was *χ*^2^ = 217.617, df = 50, *p* < 0.001, RMESEA = 0.077, CFI = 0.960, and SRMR = 0.043, and that for the four-factor model was *χ*^2^ = 213.758, df = 48, *p* < 0.001, RMESEA = 0.078, CFI = 0.960, and SRMR = 0.043. These values meet the cutoff criteria.

**Table 1:** Fit indices for the confirmatory factor analysis.

	*χ* ^2^	df	RMSEA	SRMR	CFI
One-factor	487.621	54	0.121	0.052	0.895
Four-factors	213.758	48	0.078	0.043	0.960
Four first-order factors and 1 second-order factor	217.617	50	0.077	0.043	0.960

RMSEA, Root mean square error of approximation; SRMR, Standardized root mean square residual; CFI, Comparative fit index.

**Figure 1:**
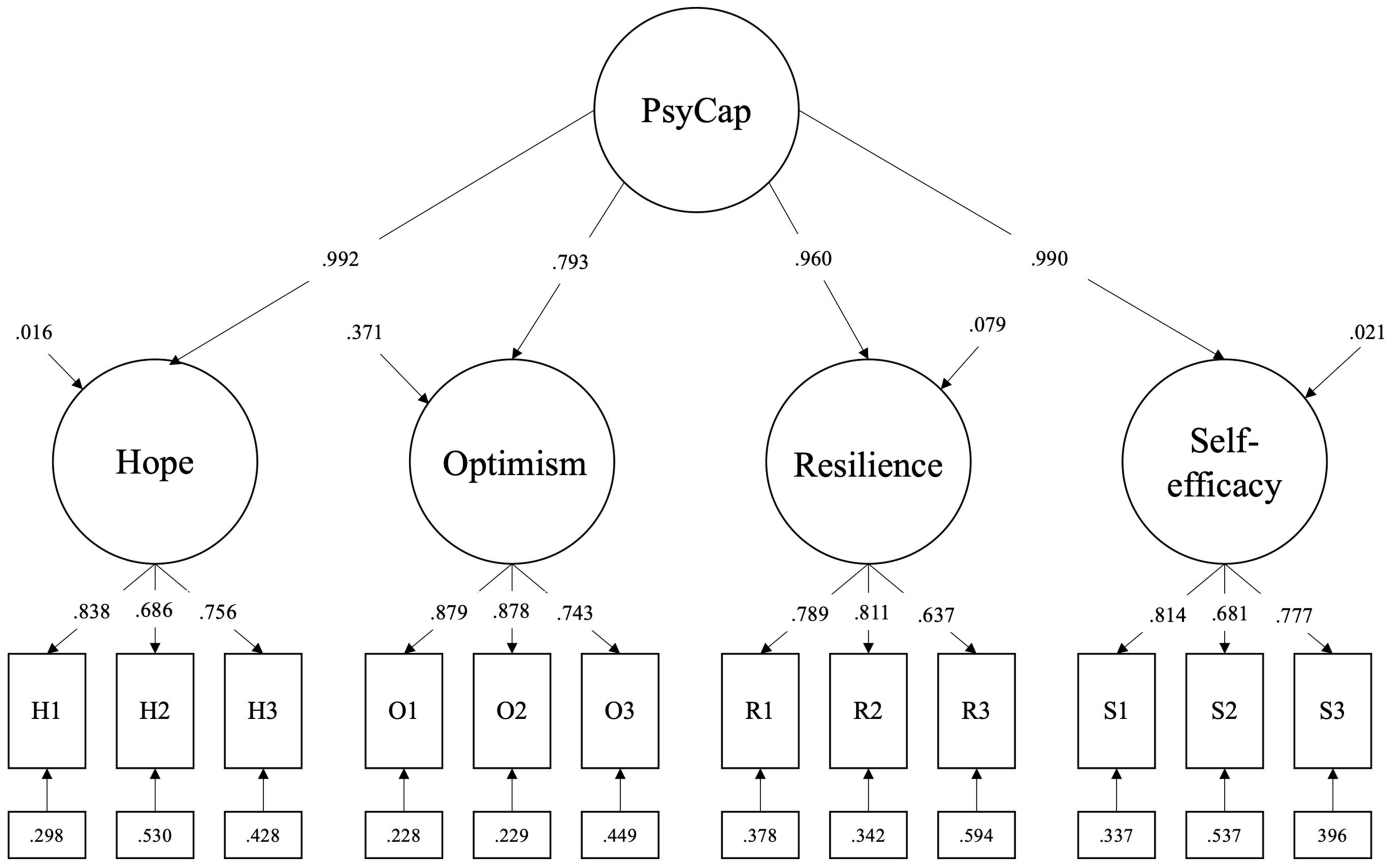
Results of confirmatory factor analysis in the model of the previous study.

Table 2 shows standardized factor loadings for each model. Further, Figure 1 presents factor loadings for the second-order factor models. The factor loadings were high and positive in both models. Based on the analysis results and previous research model (Dudasova et al., 2021), the second-order model was accepted.

**Table 2:** Factor loadings obtained by confirmatory factor analysis.

	*M*	SD	Four first-order factors and					Four-factor				One-factor
			1 second-order factor									
			Hope	Op	Resi	SE	PC	Hope	Op	Resi	SE	
If I should find myself in a jam, I could think of many ways to get out of it.	3.215	1.183	0.838					0.832				0.812
Right now, I see myself as being pretty successful.	2.902	1.308	0.686					0.693				0.701
I can think of many ways to reach my current goals.	3.445	1.206	0.756					0.754				0.742
I am looking forward to the life ahead of me.	3.565	1.358		0.879					0.880			0.742
The future holds a lot of good in store for me.	3.298	1.272		0.878					0.878			0.738
Overall, I expect more good things to happen to me than bad.	2.98	1.235		0.743					0.741			0.699
I consider myself to be able to stand a lot, and I am not easily discouraged by failure.	3.406	1.313			0.789					0.791		0.746
I believe that coping with stress can strengthen me.	3.118	1.209			0.811					0.811		0.773
After serious life difficulties, I tend to quickly bounce back.	3.443	1.222			0.637					0.634		0.637
I am confident that I could deal efficiently with unexpected events.	3.152	1.203				0.814					0.819	0.785
I can solve most problems if I invest the necessary effort.	3.215	1.183				0.681					0.675	0.691
I can remain calm when facing difficulties because I can rely on my coping abilities.	2.902	1.308				0.777					0.779	0.753
Hope							0.992		.			
Optimism							0.793					
Resilience							0.960					
Self-efficacy							0.990					

Op, Optimism; Resi, Resilience; SE, Self-efficacy; PC, PsyCap.

Cronbach’s *α* was 0.93 PsyCap, 0.790 for hope, 0.868 for optimism, 0.779 for resilience, and 0.792 for self-efficacy. Further, *ω*_ho_ was 904 for PsyCap, and *ω* was.813 for hope, 0.879 for optimism, 0.797 for resilience, and.812 for self-efficacy. Sufficient internal consistency was demonstrated for PsyCap and its subdimensions.

### 3.2. Measurement invariance

Table 3 presents results of the measurement invariance analysis. First, we examined measurement invariance for employees versus students. CFA was conducted separately for employees and students, resulting in an improper solution in the analysis for employees. Therefore, we simplified the model by deleting one item from hope (“Right now, I see myself as being pretty successful”). Goodness of fit of the simplified model was acceptable for both employees (*χ*^2^ = 68.561, df = 40, *p* < 0.001, RMESEA = 0.051, CFI = 0.986, and SRMR = 0.036) and students (*χ*^2^ = 123.859, df = 40, *p* < 0.001, RMESEA = 0.050, CFI = 0.953, and SRMR = 0.005). Next, measurement invariance was examined for this model, and construct invariance, metric invariance, and scalar invariance were supported.

**Table 3:** Fit indices for measurement invariance tests and results of model comparison.

	*χ* ^2^	df	RMSEA	SRMR	CFI	ΔRMSEA	ΔCFI	Decision
Students versus Employees								
Model 1. Configural	189.597	80	0.069	0.040	0.972			
Model 2. First-order metric	200.909	87	0.067	0.042	0.972	−0.002	0.002	Accept
Model 3. First-and second-order metric	208.116	90	0.066	0.047	0.971	−0.001	0.005	Accept
Model 4. First-order scalar	224.41	97	0.065	0.048	0.970	−0.001	0.001	Accept
Model 5. First-and second-order scalar	236.955	100	0.066	0.049	0.968	0.001	0.001	Accept
Men versus Women								
Model 1. Configural	284.164	100	0.079	0.043	0.957			
Model 2. First-order metric	298.877	108	0.077	0.046	0.958	−0.002	0.003	Accept
Model 3. First-and second-order metric	302.039	110	0.076	0.046	0.958	−0.001	0.000	Accept
Model 4. First-order scalar	316.603	119	0.073	0.048	0.958	−0.003	0.002	Accept
Model 5. First-and second-order scalar	329.275	122	0.073	0.050	0.956	0.000	0.002	Accept

RMSEA, Root mean square error of approximation; SRMR, Standardized root mean square residual; CFI, cs.

Next, measurement invariance across genders was examined. First, a CFA was performed for each gender using all items of the CPC-12R. The results showed that the goodness of fit was adequate for both men (*χ*^2^ = 165.816, df = 50, *p* < 0.001, RMESEA = 0.086, CFI = 0.951, and SRMR = 0.044) and women (*χ*^2^ = 117.153, df = 50, *p* < 0.001, RMESEA = 0.070, CFI = 0.967, and SRMR = 0.042). Second, measurement invariance was examined (Table 3). The results showed that construct invariance, metric invariance, and scalar invariance were supported.

### 3.3. Construct validity

Table 4 presents results of the correlation analysis. It provides descriptive statistics for each variable, Cronbach’s *α*, McDonald’s *ω*, and correlations with the CPC-12R. Similar to previous studies (Lorenz et al., 2016; Platania and Paolillo, 2022), CPC-12R is positively correlated with job satisfaction (*r* = 0.435; *p* < 0.001), work engagement (*r* = 0.537; *p* < 0.001), conscientiousness (*r* = 0.506; *p* < 0.001), and extraversion (*r* = 0.421; *p* < 0.001); it is negatively correlated with negative emotionality (*r* = −0.575; *p* < 0.001).

**Table 4:** Correlation of the Compound Psychological Capital Scale (CPC) with related variables.

		*M*	SD	*α*	*ω*	*r*
1	CPC-12R	3.259	0.956	0.933		
2	Job satisfaction	2.850	1.017	0.904	0.904	0.435^***^
3	Work engagement	3.033	1.340	0.960	0.966	0.537^***^
4	Conscientiousness	3.143	0.645	0.739	0.673	0.506^***^
5	Extraversion	2.635	0.727	0.744	0.744	0.421^***^
6	Negative emotionality	3.119	0.786	0.787	0.789	−0.575^***^

^***^p < 0.001.

## 4. Discussion

This study aimed to examine the reliability and construct validity of the Japanese version of the CPC-12R. Results of the analysis showed that the Japanese version of the CPC-12R is an appropriate instrument for measuring PsyCap among Japanese university students and company employees.

### 4.1. Factor structure

In this study, we compared the model of the previous study (second-order model) with an alternative model (one-factor model and four-factor model). Results of the analysis showed that the second-order model and four-factor model had a good fit. The result supporting the second-order model means that the same factor structure of the original scale (Dudasova et al., 2021) is demonstrated in the Japanese version. In addition, the reliability coefficients of PsyCap were adequate.

### 4.2. Measurement invariance

Our analysis showed the configural, metric, and scalar invariances in terms of occupations and gender. The fact that scalar invariance was upheld means that the factor structure, factor loadings and intercepts are the same across groups. Thus, the factor means can be compared across groups (Chen, 2008).

However, measurement invariance across employees and students was examined using a simplified model (with one item of hope removed). In the CFA model (common latent construct model with reflective indicators), removing one item from the measurement model does not change the meaning of the construct (MacKenzie et al., 2005). Therefore, the model with one item removed is not considered theoretically inconsistent with previous studies.

### 4.3. Examination of construct validity

Expectedly, our construct validity results showed that the Japanese version of the CPC-12R has moderate positive correlations with job satisfaction (*r* = 0.435), work engagement (*r* = 0.537), conscientiousness (*r* = 0.506), and extraversion (*r* = 0.421), as well as a negative correlation with negative emotionality (*r* = −0.575). Our results are generally consistent with previous studies showing that the CPC-12R has weak, moderate correlations with job satisfaction (*r* = 0.29–0.40), work engagement (*r* = 0.39), conscientiousness (*r* = 0.29), extraversion (*r* = 0.24), and negative emotionality (*r* = −0.49; Lorenz et al., 2016; Platania and Paolillo, 2022). In addition, our results can be interpreted as those with higher PsyCap also having higher job satisfaction and work engagement. Thus, PsyCap may be an essential factor for job performance in the Japanese context.

### 4.4. Limitations and future directions

Several limitations and challenges remain in this study. First, the data are limited to university students and employees in their 20s. Therefore, to generalize the results to other age groups and occupations, future studies should examine whether similar results can be obtained for people in their 30s and beyond.

Second, although it is desirable to have more than one researcher involved in the translation process, only one researcher and graduate student were involved in this study. Therefore, there is a limit to the quality of the translation process.

Third, the examination of measurement invariance in this study is limited to examination across different occupations and genders. Future work should examine measurement invariance across groups with different work hours, college majors, and countries.

Fourth, this study analyzed data obtained using an internet survey. The reward paid to participants was the same as that for a typical survey in Japan (e.g., Hatano et al., 2022). However, it is noted that in internet surveys, not all participants pay much attention to answering questions, as some participate in surveys only for rewards (Miura and Kobayashi, 2015). Therefore, future comparisons with questionnaire surveys are desirable.

Fifth, we did not test the predictive validity of the Japanese version of the CPC-12R. A longitudinal study may be conducted to verify whether the Japanese version of the CPC-12R can predict performance.

Finally, McDonald’s *ω* was low on the Conscientiousness scale used in the construct validity study. It may have resulted in attenuation of the correlation coefficients between the personality scale and CPC-12R. Therefore, future studies using other scales are necessary.

## 5. Conclusion

Our results showed that the CPC-12R is valid as a second-order model in Japan as in previous studies, and it has sufficient reliability and construct validity. Furthermore, the scale showed scalar invariance across students and employees, as well as gender. Thus, it can be used for comparison between these groups. This scale can thus be used to advance PsyCap research in Japan.

## Data availability statement

The raw data supporting the conclusions of this article will be made available by the authors, without undue reservation.

## Ethics statement

We obtained approval from Osaka Prefecture University’s ethics committee before conducting the survey.

## Author contributions

MI was responsible for the data analysis and writing the manuscript draft. KH guided the analysis and helped draft and was involved in important manuscript revisions. ST and JN were involved in important revisions of the manuscript. All authors contributed to the article and approved the submitted version.

## Funding

This research was supported by Dentsu Scholarship Foundation, Tokyo, Japan [Research fund to Rikkyo University 2020–2022].

## Conflict of interest

The authors declare that the research was conducted in the absence of any commercial or financial relationships that could be construed as a potential conflict of interest.

## Publisher’s note

All claims expressed in this article are solely those of the authors and do not necessarily represent those of their affiliated organizations, or those of the publisher, the editors and the reviewers. Any product that may be evaluated in this article, or claim that may be made by its manufacturer, is not guaranteed or endorsed by the publisher.
