# Supplementary material for: Validation of the Japanese version of the revised version of the compound psychological capital scale (CPC-12R)
Source: Front Psychol. 2023 Jan 18;13:1053601. doi: 10.3389/fpsyg.2022.1053601 (PMC9896786; doi:10.3389/fpsyg.2022.1053601)
Supplement: Supplementary file 1 [file Table_1.docx]

Supplementary Material for Validation of the Japanese Version of the Revised Version of the Compound Psychological Capital Scale
(CPC-12R)

**HOPE**

- If I should find myself in a jam, I could think of many ways to get out of it: 窮地に陥ったとしても、そこから抜け出すための方法をたくさん考えることができる。
- Right now, I see myself as being pretty successful: 現時点では、自分はかなり成功していると思う
- I can think of many ways to reach my current goals: 今の目標を達成するために、いろいろな方法を考えることができる

**OPTIMISM**

- I am looking forward to the life ahead of me: これからの人生を楽しみにしている
- The future holds a lot of good in store for me: 未来にはたくさんの良いことが待っている
- Overall, I expect more good things to happen to me than bad: 全体的に見て、悪いことよりも良いことの方が多く起こると思っている

**RESILIENCE**

- I consider myself to be able to stand a lot, I am not easily discouraged by failure: 多くのことに耐えることができ、失敗しても簡単には落胆ない
- I believe that coping with stress can strengthen me: ストレスに対処することで自分は強くなり得ると信じている
- After serious life difficulties, I tend to quickly bounce back: 人生で大きな困難に遭遇した後も、すぐに立ち直ることができる

**SELF-EFFICACY**

- I am confident that I could deal efficiently with unexpected events: 予想外の出来事があっても、効率的に対処できる自信がある
- I can solve most problems if I invest the necessary effort: 必要な努力をすれば、ほとんどの問題を解決できる
- I can remain calm when facing difficulties because I can rely on my coping abilities: 困難に直面しても冷静でいられるのは、自分の対処能力に頼ることができるから

The Japanese translation of the instruction is following: 以下のそれぞれの項目は普段のあなたの考え、態度について、どの程度あてはまりますか。最もあてはまるものをそれぞれ一つずつ選んでください。「1. 全くあてはまらない」〜「6. とてもあてはまる」
